# Thank you to the *Journal of the Medical Library Association* reviewers in 2018

**DOI:** 10.5195/jmla.2019.658

**Published:** 2019-04-01

**Authors:** Katherine G. Akers

**Affiliations:** Editor-in-Chief, *Journal of the Medical Library Association*, JMLA@journals.pitt.edu

## Abstract

The *Journal of the Medical Library Association (JMLA)* sincerely thanks the 210 peer reviewers in 2018.

The *Journal of the Medical Library Association (JMLA)* sincerely thanks the 210 reviewers in 2018 who helped vet and improve the quality of work published in our journal.

The *JMLA* is always looking to expand our pool of reviewers who can critically comment on any topic of research or practice in health sciences librarianship. If you are interested in serving as a peer reviewer for the *JMLA,* please send your CV to the editor-in-chief at jmla@journals.pitt.edu.

## 2018 JOURNAL OF THE MEDICAL LIBRARY ASSOCIATION REVIEWERS

Lisa M. Acuff, AHIP

Nancy E. Adams

Kendra Albright

Kristine M. Alpi, AHIP

Katherine Anderson

Katelyn Angell

Nell Aronoff

Marie T. Ascher

Elena Azadbakht

Michael Bales

Becky Baltich Nelson

Ann Barrett

Jill Barr-Walker

Michelle B. Bass, AHIP

Danielle Becker

Tanja Bekhuis, AHIP

Donna R. Berryman, AHIP

Skye Bickett, AHIP

Lindsay E. Blake, AHIP

Amy Blevins

Catherine Boden

Sarah Bonato

John Alexander Borghi

Jill Boruff, AHIP

Patricia Bradley, AHIP

Wichor Bramer

Ellen Brassil, AHIP

Riley Brian

Stewart Brower, AHIP

Janis Brown, AHIP

Nicole Capdarest-Arest, AHIP

Alexander J. Carroll, AHIP

Tallie Casucci

Susan Cavanaugh

Thane Chambers

Amy Chatfield

Marina Chilov

Keith W. Cogdill, AHIP

Marisa Conte, AHIP

I. Diane Cooper, AHIP

Jill Crawley-Low

Andrew Creamer

John W. Cyrus

Nicole Dalmer

Prudence W. Dalrymple, AHIP, FMLA

Ariel Deardorff

Sandy De Groote, AHIP

Basia Delawska-Elliott, AHIP

Frances Delwiche

Robin Desmeules

Jennifer Dinalo

Daniel Dollar

Amy Donahue

Kathel Dunn

Martha Earl, AHIP

Erin R. B. Eldermire

Yamila El-Khayat

Keith Engwall, AHIP

Mette Eriksen

Julia Esparza, AHIP

Alison Farrell

Lisa Federer, AHIP

Susan Fowler

Suzanne Fricke, AHIP

Emily Ginier

Dean Giustini

Emily J. Glenn

Abigail Goben

Alexandra Gomes, AHIP

Xan Y. Goodman, AHIP

Sally Gore

Kelsey Grabeel, AHIP

Vera Granikov

Charles J. Greenberg, AHIP

Xiaomei Gu

Jean Gudenas, AHIP

Karen Gutzman

Gali Halevi

Laura Hall

Rosie Hanneke, AHIP

Gale G. Hannigan, AHIP

Teresa Hartman

R. Brian Haynes

Margaret Henderson, AHIP

Elizabeth G. Hinton, AHIP

Toni Hoberecht, AHIP

Heather N. Holmes, AHIP

Allison M. Howard, AHIP

Carol L. Howe

Shanda Hunt

Rebecca Jacob

Sarah T. Jewell

Phill Jo, AHIP

Emily M. Johnson, AHIP

Timothy Johnson

Claire B. Joseph, AHIP

Jill R. Kavanaugh, AHIP

Susan Keller

Erin E. Kerby

Andrea M. Ketchum, AHIP

Jessica Kilham

Sujin Kim

Elizabeth J. Kiscaden, AHIP

Stephen Kiyoi, AHIP

Mary Lou Klem

Amy C. Knehans, AHIP

Peter Kokol

Julie Kosteniuk

Michelle Kraft, AHIP

Gretchen Kuntz

Leena Lalwani

Jennifer Langford

Mariana Lapidus

Janna C. Lawrence, AHIP

Mê-Linh Lê, AHIP

Cedric Lefebvre

Noah Lenstra

Len Levin, AHIP

J. Michael Lindsay, AHIP

Anne M. Linton, AHIP

Eddie Loh

Diane Lorenzetti

Tara R. Malone

Derek Marshall

Heather J. Martin, AHIP

Lisa Mastin

Sarah McClung

Karen McElfresh, AHIP

Sandra McKeown

Elizabeth McMunn-Tetangco

Misa Mi, AHIP

Elizabeth Moreton

Martin Morris

Whitney Mortensen

Bethany Myers, AHIP

Robin Naughton

Joey Nicholson

Tyler Nix

Hannah Norton, AHIP

Melanie J. Norton

Kelly O’Brien

Will Olmstadt, AHIP

Rob O’Riley

Erica Owusu

Jessica Page

Janet Papadakos

Ninfa Pena-Purcell

Robert Perret

Gerald J. Perry, AHIP, FMLA

Keith Pickett

JJ Pionke

T. Scott Plutchak, AHIP, FMLA

Kimberly R. Powell

Zahra Premji

Katherine Prentice, AHIP

Alexandria Quesenberry

Rebecca Raszewski, AHIP

Rebecca Raworth

Kevin Read

Robyn Reed, AHIP

Melissa L. Rethlefsen, AHIP

Rebecca Reznik-Zellen

Morwenna Rogers

Stephanie Roth, AHIP

Pamela Royle

Sarah Safranek

Megan Sapp Nelson

Cathy Sarli, AHIP

Nancy Schaefer, AHIP

Cynthia Schmidt

Carolyn Schubert

Stephanie J. Schulte

Diane G. Schwartz, FMLA

Barbara Sen

Carol Shannon

Robert M. Shapiro II

Chen Su-may Sheih

Jean P. Shipman, AHIP, FMLA

Mary Shultz

Shannon Sibbald

John Siegel, AHIP

Mary Simons

Angela Spencer, AHIP

Elizabeth Stellrecht

Sean Stone

Alisa Surkis

Stephanie M. Swanberg, AHIP

Natalie Tagge

Nancy Tannery

Michele R. Tennant, AHIP

Marilyn G. Teolis, AHIP

Nicole Theis-Mahon, AHIP

Annie Thompson

JoLinda L. Thompson, AHIP

Whitney A. Townsend

Natalia Tukhareli

Christine Urquhart

Jim Van Loon

Emily Vardell

Matt Vassar

Kathryn Vela, AHIP

Megan von Isenburg, AHIP

Amanda Wanner, AHIP

Carol Watwood, AHIP

Terrie R. Wheeler

Judith Wiener

Christina L. Wissinger

Joe Wu

Lin Wu, AHIP

Jingfeng Xia

Yan Zhang

